# The Surface Charge of Polymer-Coated Upconversion Nanoparticles Determines Protein Corona Properties and Cell Recognition in Serum Solutions

**DOI:** 10.3390/cells11223644

**Published:** 2022-11-17

**Authors:** Liuen Liang, Arun V. Everest-Dass, Alexey B. Kostyuk, Zahra Khabir, Run Zhang, Daria B. Trushina, Andrei V. Zvyagin

**Affiliations:** 1 MQ Photonics Centre, Macquarie University, Sydney, NSW 2109, Australia; 2 Laboratory of Optical Theranostics, Nizhny Novgorod State University, 603950 Nizhny Novgorod, Russia; 3 Australian Research Council Industrial Transformation Training Centre for Facilitated Advancement of Australia’s Bioactives (FAAB), Macquarie University, Sydney, NSW 2109, Australia; 4 Australian Institute for Bioengineering and Nanotechnology, The University of Queensland, St. Lucia, QLD 4072, Australia; 5 Institute of Molecular Theranostics, Sechenov First Moscow State Medical University, 119991 Moscow, Russia; 6 Federal Scientific Research Centre “Crystallography and Photonics” of Russian Academy of Sciences, 119333 Moscow, Russia; 7 Shemyakin-Ovchinnikov Institute of Bioorganic Chemistry of the Russian Academy of Sciences, 117997 Moscow, Russia

**Keywords:** protein corona, protein cloud, upconversion nanoparticles, polymer coating, cellular uptake, cytotoxicity

## Abstract

Applications of nanoparticles (NPs) in the life sciences require control over their properties in protein-rich biological fluids, as an NP quickly acquires a layer of proteins on the surface, forming the so-called “protein corona” (PC). Understanding the composition and kinetics of the PC at the molecular level is of considerable importance for controlling NP interaction with cells. Here, we present a systematic study of hard PC formation on the surface of upconversion nanoparticles (UCNPs) coated with positively-charged polyethyleneimine (PEI) and negatively-charged poly (acrylic acid) (PAA) polymers in serum-supplemented cell culture medium. The rationale behind the choice of UCNP is two-fold: UCNP represents a convenient model of NP with a size ranging from 5 nm to >200 nm, while the unique photoluminescent properties of UCNP enable direct observation of the PC formation, which may provide new insight into this complex process. The non-linear optical properties of UCNP were utilised for direct observation of PC formation by means of fluorescence correlation spectroscopy. Our findings indicated that the charge of the surface polymer coating was the key factor for the formation of PC on UCNPs, with an ensuing effect on the NP–cell interactions.

## 1. Introduction

The design, production, and applications of biofunctional nanoparticles (NPs) are at the heart of the field of nanomedicine, which paves way for modern personalized medicine. Compared with bulk materials, nanoparticles possess several unique characteristics, such as controllable nanometer size and shape, large surface-area-to-volume ratio, and adjustable surface chemistry, allowing to assemble cargo-loading vectors for theranostics applications [1,2,3]. Besides, the surface of NPs can be functionalized with targeting molecules to enable navigation in complex in vivo environment and specific binding to cells [4,5,6]. The state-of-the-art theranostics nanocomplexes make use of the excellent contrast properties of quantum dots, gold, and iron oxide nanocrystals. These prominent characteristics of NPs have demonstrated promise for the diagnosis and treatment of disease at an early stage.

Among a variety of NPs useful for theranostics applications, lanthanide-doped upconversion nanoparticles (UCNPs) have gained considerable attention over the last two decades due to their unique optical properties [3]. Unlike the existing fluorescent contrast agents (organic dyes and quantum dots), UCNPs are photoluminescent nanomaterials that can absorb several near-infrared (NIR) low-energy photons in order to emit the higher-energy anti-Stokes photons. This upconversion excitation–emission process endows UCNPs with significant advantages in biomedical imaging. These include an exceptional blinking-free photostability, superior contrast with suppressed background, deep-tissue imaging capability, wavelength-controllable narrow spectral bands, enabling multiplexed imaging, and long emission lifetime (100−1000 µs), allowing for time-gated imaging with suppressed background [7,8,9]. Moreover, tunable UCNP emission over a broad range of wavelengths can be engineered. This allows UCNPs to be used as a versatile energy transducer to convert near-infrared (NIR) light into ultraviolet (UV), visible light in order to initiate photoreactions at a sub-centimeter depth in biological tissue. Additionally, bare and polyethylene glycol-coated (PEG) lanthanide-doped UCNPs exhibit negligible cytotoxicity at concentrations lower than 0.05 and 1 mg/mL, respectively [10,11]. It is known that hemolysis to rabbit red blood cells was less than 2% at concentrations of bare UCNPs up to 20 mg/L [11]. No negative changes to the animal weight or clear signs of damage or inflammation in the key organs of mice treated with 15 mg/kg of PEGylated UCNPs have been observed for at least 30 days after an injection, indicating high biocompatibility of these NPs [10]. Polymer-coated UCNPs can be partially excreted, and the others remain in the mononuclear phagocyte system for three months, showing no overt toxicities [12].

The demonstrated application scope of UCNP is broad, including multi-model cell and animal imaging, biosensing, drug/gene delivery, photodynamic therapy, and photothermal therapy [13,14]. The latest results showed that UCNPs could provide high-quality images using time-gated detection to monitor viral infection in vitro and in vivo [15], demonstrating potential of UCNPs in the context of the SARS-CoV-2 pandemic.

Harnessing UCNP interactions with cells is a gateway to these applications, where the surface characteristics of UCNPs have been generally consented to play the key role. However, realistic cellular context, such as complete culture medium, adds an extra dimension to the interaction scenario due to the swift nonspecific binding of proteins to UCNPs, before they encounter cells. It has recently been recognised that NPs entering biological systems are quickly covered by biomolecules (e.g., plasma proteins) in order to form a protein corona (PC) layer [16,17].

Based on the affinity, binding, and dissociation rate, protein corona are classified into hard and soft PC [18,19]. The hard PC is formed within several seconds, its most surface proximal layer is characterised by longer residence time, more stable composition, and modest protein exchange with a protein-abundant environment. The soft PC is formed following the completion of hard corona formation, reaching equilibrium with the hard PC and environment within several minutes to several hours. The soft PC is more distal from the NP surface and more susceptible to exchange and interaction with other proteins in the biological environment. Some authors distinguish the third part–interfacial PC [20]. Since PC formation is a dynamic process, complex surface–protein and protein–protein interactions are not only dependent on the NP properties, but also on the biological environment [21]. The system of protein-modified NPs forms a unique biological identity of NPs that determines their interactions with biological systems. PCs are formed on most NPs regardless of their composition and surface properties, while NPs with profound antifouling properties and reduced adsorption of proteins are reportedly rare [22,23,24]. As a result, the physiochemical properties of NPs, such as size, shape, surface charge, hydrophilicity, and aggregation state, are altered dramatically. Accordingly, the biological properties of NPs in terms of toxicity, cellular recognition, cellular uptake, blood circulation lifetime, tumour accumulation, and biodistribution are also affected. For example, the PC has been shown to reduce NP adhesion to cell membrane and mitigate the associated cytotoxic effects, which the NP would otherwise exert [25,26,27]. The precoating of NPs with the most abundant plasma protein, albumin, has been demonstrated to halt further protein adsorption, decrease complement activation, prolong blood circulation time, and reduce NP toxicity [28,29]. On the other hand, the PC can screen targeting molecules attached to the surface of NPs and decrease their specificity to target cells [16,30]. Although an understanding NP PC and its influence on the biological functions of NPs is under the research spotlight, PC formation on UCNPs and its effects in cell culture medium or in body fluids has not been sufficiently addressed.

In this work, we aimed to address the formation of protein corona on UCNPs in serum-supplemented cell culture media and the influence of the PC on the interaction between incubated UCNPs and cells. A comparative study was performed in order to evaluate the UCNP surface charge effect on PC formation in complete cell culture medium. To this aim, UCNPs were functionalised with polyethyleneimine (PEI) and polyacrylic acid (PAA) in order to render NPs with positive and negative surface charges, respectively. PC formed on these NPs was visualised using TEM and negatively stained samples. The change of the UCNP physiochemical properties due to serum proteins in the cell culture medium were also characterised in terms of the hydrodynamic size and ζ-potential. Using high-performance liquid chromatography and electrospray ionisation mass spectrometry (LC-ESI-MS), a comprehensive proteomics investigation for the identification and analysis of UCNP-PEI- and UCNP-PAA-associated hard coronas was performed. The PC effect on the cytotoxicity and cell association (cell binding and uptake) of UCNPs was investigated and compared with that of bare UCNPs.

In order to demonstrate the utility of photoluminescent nanomaterials with nonlinear optical properties, we carried out direct observation of PC formation on polymer-coated UCNPs in protein solutions by means of fluorescence correlation spectroscopy. The use of the UCNP photoluminescent signal elicited by the multiphoton excitation helped to suppress optical scattering background due to serum proteins. Since the UCNP signal was acquired from a sub-femtolitre volume containing several NPs, the particle size distribution (PSD) was sampled more accurately than that acquired by dynamic light scattering (DLS) from an ensemble of many NPs. It is well known that DLS approaches tend to overestimate the contribution from the large-size fraction of the PSD. Therefore, the demonstrated fluorescence correlation spectroscopy of the UCNP emission provided an alternative approach of observation of PC formation in serum solutions, which can be implemented as direct, non-destructive, and potentially more accurate to sample colloidal PSD.

## 2. Materials and Methods

Branched polyethyleneimine (PEI, Mw = ~25,000), poly(acrylic acid) (PAA, Mn = ~130,000), cyclohexane (99.5%), nitrosyl tetrafluoroborate (NOBF_4_, 95%), dichloromethane (≥99%), dimethylformamide (DMF), toluene (≥99.5%), ethanol (≥99.8%), *N*-(3-dimethylaminopropyl)-*N*’-ethylcarbodiimide hydrochloride (EDC∙HCl), *N*-hydroxysulfosuccinimide sodium salt (Sulfo-NHS), tungstophosphoric acid (PTA), thiazolyl blue tetrazolium bromide (MTT), and dimethyl sulfoxide (DMSO), paraformaldehyde (PFA), Dulbecco’s Modified Eagle’s medium (DMEM), fetal bovine serum (FBS), nitric acid (70%), oleic acid, formic acid, fluorescein isothiocyanate conjugated lectin from triticum vulgaris (wheat) (WGA-FITC), 4′6-diamidino-2-2phenylindole (DAPI), trypsin, dithiothreitol (DTT), iodoacetamide (IAA) were purchased from Sigma-Aldrich (St. Louis, MO, USA). Methoxy-poly(ethylene-glycol)-amine (PEG-NH_2_) was purchased from Lyasan Bio (Arab, AL, USA). Phosphate buffered saline (PBS, pH = 7.4) and penicillin-streptomycin (P/S, 10,000 U/mL) were purchased from Life Technologies (Australia). Thermo-Scientific Pierce Micro bicinchoninic acid (BCA) protein assay kit was purchased from Thermo Fisher Scientific (Australia). All reagents were used as received without further purification.

Transmission electron microscopy (TEM) images were obtained using a Philips CM10 transmission electron microscope (Philips, Eindhoven, the Netherlands) at 100 kV. For negative staining, nanoparticles on formvar-coated copper grids were incubated with 5 µL of 2% PTA aqueous solution (pH = 7.0) for 30 s at room temperature. The excess staining solution was drained off, and the samples were dried and observed under TEM. Upconversion photoluminescence spectra were recorded by using a Fluorolog-Tau3 spectrofluorometer (Horiba scientific, Kyoto, Japan). Dynamic light scattering (DLS) and ζ-potential were determined using a Nano ZS90 Zetasizer (Malvern, UK). NPs were diluted with distilled water for the measurements. Fourier transform infrared (FTIR) spectra were acquired using a Nicolet iS10 FTIR spectrometer (Thermo Fisher Scientific). Thermal gravimetric analysis (TGA) was performed using a TGA/DSC 1 STARe System (Mettler Toledo, Columbus, OH, USA).

### 2.1. Preparation of UCNP-PEI and UCNP-PAA

Hexagonal-phase UCNPs (NaYF_4_: 18% Yb, 2% Er) were prepared via the solvothermal decomposition method as described in our previous work [31]. NPs of different batches were mixed to obtain ~2 g of homogenized oleate-capped UCNPs (UCNP-OA) for subsequent surface modification with polymers. To modify UCNPs with PEI and PAA, oleate ligands on UCNP surface were first removed with NOBF_4_. Typically, 10 mL of UCNP-OA cyclohexane suspension (5 mg/mL) was mixed with 10 mL of NOBF_4_ dichloromethane solution (1.17 mg/mL) in a sealed flask and kept under vigorous stirring at room temperature overnight. The resulting NOBF_4_-capped UCNPs (UCNP-NOBF_4_) were pelleted by centrifugation, and the supernatant was discarded. The precipitated nanoparticles were redispersed in DMF flocculated with the mixture of toluene and cyclohexane. After centrifugation, 5 mL of DMF was added again in order to disperse the nanoparticles. For the synthesis of UCNP-PAA, 5 mL of PAA DMF solution (30 mg/mL) was added to the UCNP-NOBF_4_ suspension. The mixture was heated to 80 °C under stirring for 3 h in a sealed flask. The UCNP-PAAs were then collected using centrifugation and washed three times with ethanol and three times with distilled water. The final product was dispersed in distilled water for storage. UCNP-PEIs were prepared using the same protocol except an addition of 25 mL of PEI ethanol solution (4 mg/mL) instead of that of PAA. The PEI ligand exchange reaction was conducted at room temperature for 24 h. For the hydrodynamic size investigation using FCS, Tm-doped UCNPs (NaYF_4_: 20% Yb, 8% Tm) were prepared, and the NP surface was modified with PEI and PAA via methods described elsewhere [32].

PEG-NH_2_ was grafted to UCNP-PAA in order to obtain PEG-modified UCNP (UCNP-PEG) via chemical bonding. For this, 1 mg of UCNP-PAA were dispersed with 1 mL distilled water (pH = 5.5) and the carboxylic groups of PAA were activated by 0.4 mg of EDC·HCl and 1.1 mg sulfo-NHS. After 30 min of incubation at room temperature, the nanoparticles were centrifuged and washed with distilled water three times and then dispersed with 200 µL of PBS (pH = 7.4) via sonication in cold condition. A total of 200 µL of PBS containing 1.5 mg of PEG-NH_2_ was added in followed by incubation on a rotary shaker at 4 °C overnight. The unreacted PEG-NH_2_ was removed by centrifugation and the UCNP-PEG particles were purified by washing three times with distilled water.

### 2.2. Formation of Hard Protein Corona on UCNPs

Before incubating with UCNPs, complete cell culture medium (DMEM supplemented with 10% FBS and 1% P/S) was filtered through a 0.22 µm membrane filter in order to remove clustered proteins. To form a PC on UCNPs, 1 mg UCNPs were dispersed with 1 mL complete cell culture medium. The suspension was sonicated under cold condition for 30 s, and then shaken in a rocking incubator at 37 °C for different time intervals. The UCNP-PC colloids were centrifuged at 10,000× *g* for 7 min and redispersed in PBS. The suspension was transferred to a new vial. The same washing steps with centrifugation were repeated three times to remove unbound or loosely bound proteins (soft and interfacial protein corona), so that the UCNPs were coated with only a hard corona. Finally, the UCNP-PCs were dispersed with 1 mL PBS. Prior to all characterisation and testing, UCNP-PCs were freshly prepared. UCNP-PC samples were prepared by incubating polymer-modified UCNPs with complete cell culture medium for 24 h for subsequent cellular assays.

### 2.3. Characterisation of UCNP Particle Size Distribution via Fluorescence Correlation Spectroscopy

Fluorescence (photoluminescence) correlation spectroscopy (FCS) measurements were performed using an inverted laser-scanning confocal microscope LSM 710 NLO (Carl Zeiss, Germany) equipped with a time-correlated single photon counting (TCSPC) system (Simple-Tau 152, Becker&Hickl GmbH, Berlin, Germany). UCNPs were photoexcited by a Chameleon Vision II (Coherent, Santa Clara County, CA, USA) Ti:Sa femtosecond laser operated at a wavelength of 975 nm and focused by a water immersion objective (C-Apochromat 63×/1.2 w). The emitted light was collected by the same objective and sent through a dichroic mirror (ZT1064rdc-sp, Chroma, Irvine, CA, USA), a short-pass emission filter (FF01-890/SP, Semrock, New York, NY, USA) to the non-descanned port of the microscope. The light was then split by a dichroic beam splitter (FF552-Di02, Semrock, West Henrietta, NY, USA), passed through a band-pass filter (FF01-466/40, Semrock, New York, NY, USA) and detected by a hybrid detector (HPM-100-40, Becker&Hickl). Photons were discretely recorded by an SPC-150 card (Becker&Hickl) in a single-photon counting mode and sampled in 100 ps time bins. All measurements were performed in a temperature-controlled laboratory at 21 °C using an average excitation power of ~ 840 μW as measured at the sample. The focal plane was set to as high as 240 μm from the cuvette bottom in order to avoid a strong signal from the precipitated UCNP aggregates inside the solution. A total of 50–60 autocorrelation curves (time interval, 10 s) were acquired for each sample. The diffusion time (*τd*) and number of particles in the acquisition volume (*N*) were determined by fitting each autocorrelation function. A control experiment in distilled water was carried out for each series of FCS measurements. The effect of BSA on the solution viscosity was examined using a linear approximation based on the intrinsic viscosity of the protein provided by the manufacturer (η = 4.13 cm^3^/g) [33].

### 2.4. Quantification of Protein Adsorbed on UCNPs

The kinetics of a hard PC on UCNPs were quantified by micro-BCA assay according to the manufacturer’s protocol. UCNP-PC was formed by incubating UCNPs in complete cell culture medium for 10 min, 30 min, 1 h, 2 h, 4 h, and 24 h followed by 3 cycles of centrifugation and redispersion as previously described. A total of 25 µL of UCNP-PC suspensions (1 mg/mL) were pipetted into 5 replicate wells of a 96-well plate. Then, 200 µL of BCA reagent was added in each well. The mixture was shaken on a rocking platform for 5 min and then incubated at 60 °C for 60 min in dark conditions. After cooling to room temperature, the absorbance of the samples was measured at the wavelength 562 nm using a PHERAstar plate reader with the background absorbance subtracted. Same measurements were carried out on a series of bovine serum albumin solution with increasing concentrations (0−125 µg/mL) to plot the standard curve. Samples of UCNP-PAA in PBS and UCNP-PAA in BSA standard solutions were used as controls. The total protein concentration was calculated relative to the BSA standard.

### 2.5. Proteomics of Protein Adsorbed on UCNPs

The hard corona formed on polymer-coated UCNPs were identified using a nanoLC-ESI MS approach. Samples of the PEI- and PAA-modified UCNPs (volume, 50 μL; concentration, 1 mg/mL) in triplicates were denatured at 95 °C for 5 min and reduced (60 °C for 1 h) in 10 mM DTT. These samples were then alkylated with 25 mM IAA (30 min in dark) and digested with trypsin overnight (1 μg). The samples were centrifuged for 5 min at 14,100× *g*. The supernatant containing digested peptides was transferred into new tubes and dried. Samples were neutralised using 40 μL 0.1% formic acid prior to nanoLC-ESI-MS analysis.

A Triple TOF 6600 (AB Sciex) mass spectrometer connected to Eksigent Ultra nanoLC system (Eksigent) was used for the analysis. The digested peptide samples were chromatographically separated using a Halo C18, 160 Å, 2.7 μm, 150 μm × 10 cm analytical column. The digested sample (10 μL) was injected into a peptide trap (Michrome peptide Captrap) for pre-concentration and then desalted with 0.1% formic acid, 2% ACN, at 10 μL/min for 5 min. The peptide trap was then switched into line with the analytical column. Peptides were eluted from the column using a linear solvent gradient with steps from H_2_O:CH_3_CN (95:5; +0.1% formic acid) to H_2_O:CH_3_CN (5:95; +0.1% formic acid) at a constant flow rate (600 nL/min) over 80 min gradient. The LC eluent was analysed by a positive ion nanoflow electrospray MS in an information-dependent acquisition mode (IDA). In the IDA mode, a TOFMS survey scan was acquired (*m*/*z* 350–1500, 0.25 s), with the twenty largest multiply charged ions (counts > 200) in the survey scan being sequentially subjected to MS/MS analysis. The tandem MS/MS spectra were accumulated for 200 milliseconds (*m*/*z* 100–1500) with rolling collision energy. The generated raw data files (.wiff) were converted into mascot generic files (.mgf) using AB SCIEX CommandDriver software and submitted to Mascot (Matrix Science, London, UK) before being searched against SwissProt, another mammalian database.

The protein abundance data were calculated using normalized spectral abundance factors (NSAF) [34] with the addition of a spectral fraction of 0.5 to all spectral counts in order to compensate for null values and enable log transformation for subsequent statistical analyses. Summed NSAF values were used as a measure of the relative protein abundance. In order to identify differences in the protein abundance, the natural log NSAF values were analysed using a series of t-tests, and the significance level was set at *p* < 0.05 for all comparisons. A variance analysis (ANOVA) was also performed in order to identify proteins changing in abundance among those proteins which presented reproducibly in the three UCNP surface-modification conditions. Data processing was carried out using the Scrappy package [34].

### 2.6. Cell Culture

MDA-MB-231 human breast cancer cells were cultured in complete cell culture medium containing DMEM medium, 10% (*v*/*v*) FBS, and 1% (*v*/*v*) P/S, and maintained at 37 °C in a humidified incubator with 5% CO_2_.

### 2.7. Cytotoxicity Study

The cytotoxicity of nanoparticles for MDA-MB-231 cells were evaluated by MTT assay. MDA-MB-231 cells were plated in 96-well plates at the density of 5000 cells/well. After incubation overnight, cells were washed 3 times with PBS in order to remove residual FBS. UCNPs with or without a hard corona were dispersed with DMEM in order to obtain suspensions with concentrations 0, 12.5, 25, 50, 100, and 200 µg/mL. Cells were then incubated with UCNP suspensions for 24 h. MTT assays were conducted in order to determine cell viability with respect to the control without treatment.

### 2.8. Quantification of Cell-Associated UCNPs via Inductively Coupled Plasma Mass Spectroscopy (ICP-MS)

To measure the amount of cell-associated UCNPs, MDA-MB-231 cells were seeded into 12-well plates with a density of 10^5^ cells/well and allowed to grow until 80% confluency. Next, 1 mL of UCNPs was added to MDA-MB-231 cells at the concentrations of 2.5, 5, 10, and 20 µg/mL for incubation periods of 0.5, 1, 2, 4, 6, 12, and 24 h. Cells in DMEM without NP treatment were used as the control. The UCNP suspension was removed, and cells were rinsed 3 times with PBS in order to remove unbound UCNPs, and then harvested with trypsin for cell counting and centrifuged at 1000× *g* for 5 min to separate from growth medium. To dehydrate, cells were placed in an oven at 60 °C for 24 h. Samples were then digested with 500 µL of concentrated nitric acid (70%) for 4 h and diluted with 9.5 mL of distilled water. The yttrium content in each sample was analysed by ICP-MS. The ICP-MS results represented an average of triplicate samples, and error bars represented the standard deviation.

### 2.9. Visualisation and Analysis of Cell-Associated UCNPs via Multiphoton Microscopy

MDA-MB-231 cells were seeded at a density of 2.5 × 10^4^ cells/well onto 24-well plates with a coverslip placed at the bottom of each well. After overnight incubation, medium was discarded, and cells were washed 3 times with fresh DMEM and treated with 500 µL of UCNP or UCNP-PC suspension (10 or 20 µg/mL) in a serum-free DMEM medium. After incubation for 0.5 h to 24 h, cells were washed 3 times with PBS and fixed with 4% PFA solution. After washing with PBS 3 times, the cell membrane was stained with WGA-FITC solution, and the nuclei were stained with DAPI solution for 15 min at room temperature. Multiphoton images were acquired using a Zeiss LSM 880 laser-scanning confocal microscope equipped with a fiber-coupled continuous-wave 978-nm diode laser as the UCNP excitation source. A 40× oil objective lens was used, and images were captured and processed using ImageJ software. For image analysis, membrane-stained images were used to outline each cell. The upconversion photoluminescence from the outlined area was quantified as a UCNP signal per area and considered as a measure of the mean cell-associated UCNPs. At least 100 cells were analysed in each sample in order to obtain a statistically acceptable UCNP signal per area.

### 2.10. Statistical Analysis

Data are presented as mean ± standard deviation. Statistical analysis was performed by one-tailed paired Student’s *t*-test. Statistical significance was designated with * *p* < 0.05, ** *p* < 0.01, and *** *p* < 0.001.

## 3. Results and Discussion

### 3.1. Characterisation of Polymer-Coated UCNPs

UCNPs were synthesised as described previously using a one-pot solvothermal decomposition method and were subsequently coated with PEI or PAA to yield positively (UCNP-PEI) or negatively (UCNP-PAA) charged surfaces, respectively. TEM images of the as-produced samples showed that UCNPs were monodispersed with an average diameter of 22.8 ± 1.8 nm. The shape and physical diameter of UCNPs were not affected by the surface modification (Figure S1a). The binding of polymers to the surface of UCNPs was validated by FTIR spectroscopy (Figure S1b) and TGA analysis (Figure S1c). The observed absorption bands at 1640 and 1520 cm^−1^ in the UCNP-PEI spectrum corresponded to the stretching vibrations of N–H and C–N in PEI, respectively. The absorption bands at 1700 and 1160 cm^−1^ in the UCNP-PAA spectrum were attributed to the C=O and C–O stretching vibrations in the carboxylic groups, respectively, indicating the presence of PAA on UCNPs. The significant difference between the spectra and characteristic absorption of the functional groups confirmed the successful surface modifications of UCNPs with PEI and PAA. The TGA curve of UCNP-PEI showed a total weight loss of ~3% from 36 °C to 700 °C when the absorbed water and organic groups of the sample were completely combusted, compared to a ~5% weight loss in the sample of UCNP-PAA (Figure S1c). Furthermore, the effect of the polymer coatings on the upconversion photoluminescent properties of UCNP-PEI and UCNP-PAA were studied (Figure S1d). Both UCNP aqueous suspensions exhibited similar upconversion photoluminescent spectra under NIR excitation at 980 nm. Three dominant peaks at 525, 540, and 655 nm were observed, corresponding to the ^2^H_11/2_→^4^I_15/2_, ^4^S_3/2_→^4^I_15/2_, and ^4^F_9/2_→ ^4^I_15/2_ transitions of Er^3+^ ions. A negligible difference was detected in emission intensity between UCNP-PEI and UCNP-PAA samples (Figure S1d), indicating a similar effect of the polymer coatings on the photoluminescence conversion efficiency of UCNPs.

### 3.2. Effect of The Protein Adsorption on The Physiochemical Characteristics of Polymer-Coated UCNPs

**Figure 1:**
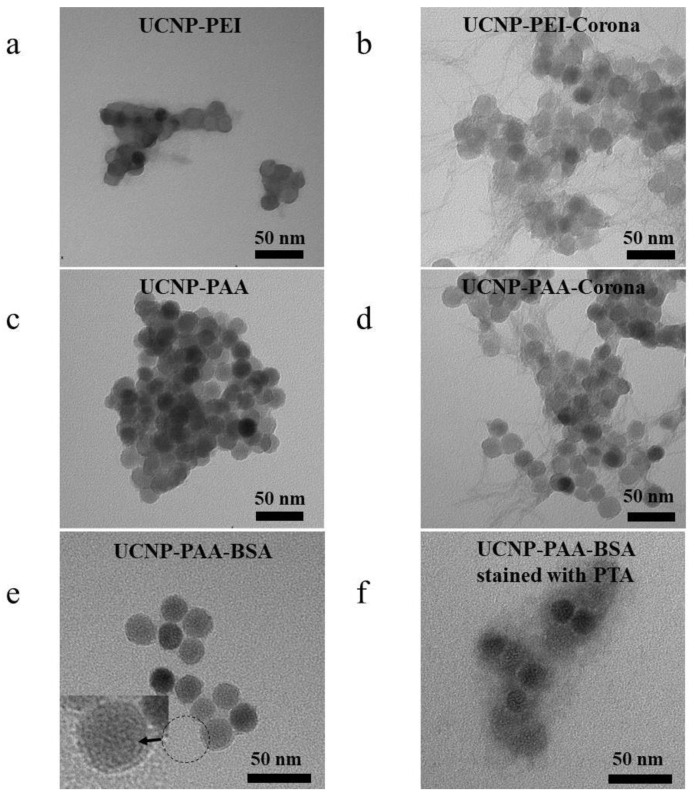
TEM images of UCNP-PEI (**a**), UCNP-PEI-PC (**b**), UCNP-PAA (**c**), UCNP-PAA-PC (**d**), UCNP-PAA-BSA corona (**e**), and UCNP-PAA-BSA corona subjected to negative staining with 2% PTA (**f**).

The presence of a hard PC on the surface of NPs pretreated with cell culture medium with 10% FBS was verified by TEM using PTA negative staining. Compared with the untreated UCNPs, hard PCs were visualised on the UCNP-PCs as a disordered protein network (Figure 1). This protein network was observed for both polymer-coated UCNPs irrespective of their surface charge. However, further investigation of UCNP-PAA incubated with 10% BSA revealed a uniform layer of protein surrounding UCNPs (Figure 1e), which was mostly observed on TEM images of UCNPs conjugated with mono-protein solutions [35,36,37]. PTA staining of the BSA coated nanoparticles revealed a protein cloud instead of a monolayer surrounding the nanoparticles (Figure 1f), indicating multilayered protein adsorption. The entangled PC patterns are clearly observable in Figure 1b,d and were probably a result of the unstable and reversible processes of PC formation in FBS [19,38,39]. The diversity of FBS proteins further contributed to the complexity of the protein adhesion. The heterogeneous charge and hydrophobic domains of FBS proteins governed the observed convoluted self-assembly in TEM images.

**Figure 2:**
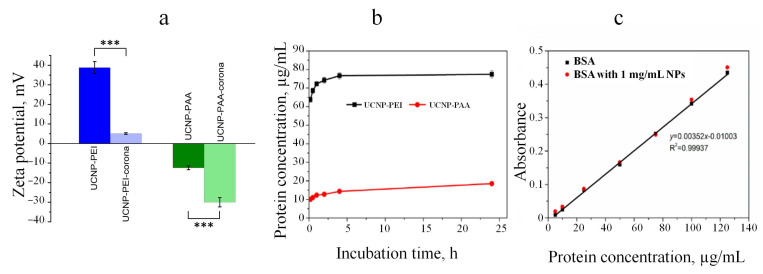
Zeta-potential measurement of polymer-coated UCNPs before and after protein corona formation (**a**). The amount of protein adsorbed on UCNP-PEI and UCNP-PAA after incubation with complete cell culture medium sampled at several timepoints quantified by BCA assay (**b**). Each value represents the mean ± standard deviation of 5 replicates. Standard absorbance–concentration curves for BSA in the presence and absence of 1 mg/mL UCNP-PAA (**c**). Statistical significance was designated with *** *p* < 0.001.

The DLS and FCS characterisation of UCNP-PEI and UCNP-PAA samples before and after PC formation are shown in Figure 2. An obvious size increase was observed for both types of UCNPs as a result of the UCNPs incubation with complete cell culture medium and FBS and this was manifested as an upshift in the particle size distribution (PSD). The protein adsorption also led to PSD broadening. As the hydrodynamic diameter of proteins in the cell culture medium was ~6.5 nm (Figure S2), the obtained mean size increase and broader PSD suggested a significant aggregation of UCNPs during PC formation. The PSD shift was noticeably larger for the PEI-modified UCNPs compared to that of PAA-UCNPs, indicating a higher degree of protein adsorption on UCNP-PEI. The higher adsorption of plasma proteins on the positively charged PEI-coated NPs compared to the negatively charged polymer-coated UCNPs was also noted for other types of NPs [18,40]. We conjecture that positively charged coatings modulate PC assembly more profoundly. 

Electrostatic force is a significant factor in determining the adsorption of proteins at the charged surface and, as a result, the PC protein composition and abundance [41]. The complete cell culture medium was enriched with serum proteins with isoelectric points (pI) below the pH (7.4) of the cell culture medium (Table S1). Thus, the abundant serum proteins generally possess a net negative charge, thereby leading to a tendency of absorbing a greater protein amount to positively charged surfaces [18,28,40]. Indeed, the zeta potential of the polymer-coated UCNPs became more negative following PC formation, which was consistent with the findings from other studies [26,27,40,42]. The zeta-potential values decreased from 38.8 ± 3.0 mV to 5.1 ± 0.4 mV and from −12.4 ± 1.0 mV to −30.0 ± 2.4 mV for UCNP-PEI and UCNP-PAA, respectively (Figure 2a). The small absolute value of the zeta potential of the UCNP-PEI-PC sample confirmed the above-mentioned aggregation of these particles.

### 3.3. Protein Corona Formation Kinetics

The hard PC was formed by incubating UCNPs with complete cell culture medium for different periods. After 3 PBS washes, hard PC remaining on the NPs surface was assayed with BCA. As shown in Figure 2b, both types of UCNPs were observed to acquire a measurable hard corona following 10 min incubation in cell culture medium. The amount of adsorbed protein was determined with the help of the preliminarily plotted calibration curves (Figure 2c). Overall, the corona protein content increased rapidly during the first 2 h of incubation, exhibiting the onset of saturation after 4 h incubation. UCNP-PEI showed approximately 4-fold more protein adhesion (77.4 µg/mL) after 24 h incubation in comparison with that of UCNP-PAA (18.5 µg/mL). The kinetic studies demonstrated that PC formation can be subdivided into two phases: firstl a tightly bound layer of proteins interacting directly with the particle surface is formed, followed by loose association with a secondary protein layer [18,29]. The duration of the first stage was less than 1 min, and it was mainly determined by the physicochemical properties of the particles. The second phase involved the adsorption of proteins onto proteins that had already been adsorbed. Figure 2 shows that several hours were required for equilibration of the PC formation process.

### 3.4. Proteomic Analysis of the Protein Coronas

**Figure 3:**
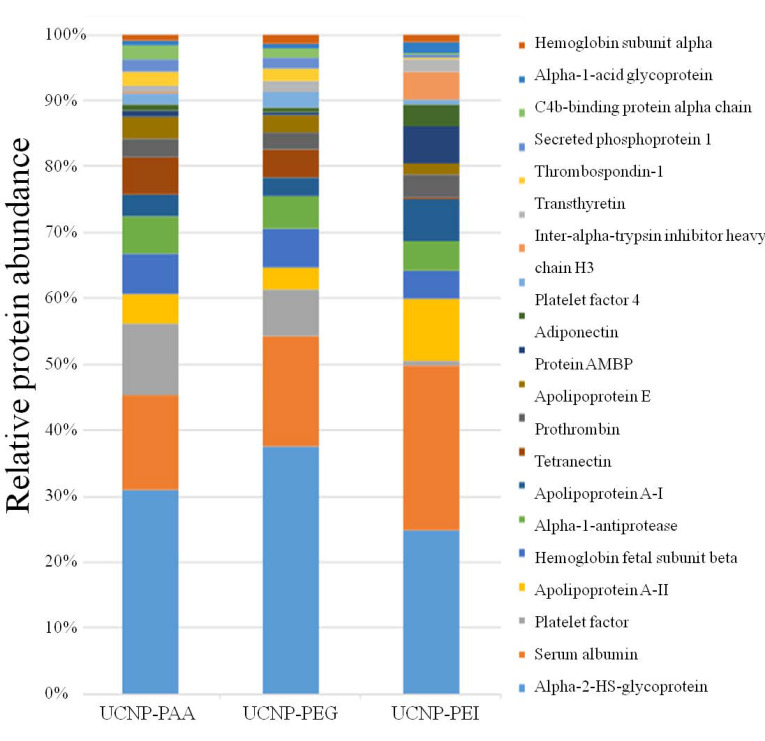
A bar chart of the relative content of 20 most abundant proteins in the PC of UNCP-PAA, UCNP-PEG, and UCNP-PEI, according to nanoLC-ESI-MS mass spectrometry analysis.

In order to identify and quantify the composition of the hard PC formed on the surface of polymer-coated UCNPs, the purified UCNP-PEI-PC, UCNP-PAA-PC, and UCNP-PEG-PC samples underwent denaturation with reducing agents, followed by enzymatic trypsin digestion in order to release proteins bound to the NPs. The mass spectrometry data showed that different classes of proteins constituted the PC of the surface-modified UNCPs. Of 68 unique proteins identified, 57 were found in common between the tested samples. The most abundant 20 PC proteins from 3 samples are presented in Figure 3. The resultant overall protein abundance was consistent with the previously reported abundance of serum proteins [18,29]. As is known, NPs injected into the bloodstream encounter the most abundant serum protein, albumin, with a concentration of 35–50 mg/mL [43,44]. It was not surprising to find that its content in the protein corona was very high. Serum albumin ranked as the second most-abundant protein in our tested samples and accounted for 15–25% of the total protein content.

The protein corona of UCNP-PEI was markedly different from that observed in UCNP-PAA. For example, although Alpha 2-HS glycoprotein was found to be the most abundant protein among the tested samples, its abundance in the UCNP-PEI corona was significantly lower than that of the UCNP-PAA and UCNP-PEG coronas. Similarly, Apolipoprotein A-I and Apolipoprotein A-II were more abundant in UCNP-PEI PC compared to UCNP-PAA and UCNP-PEG PCs.

**Figure 4:**
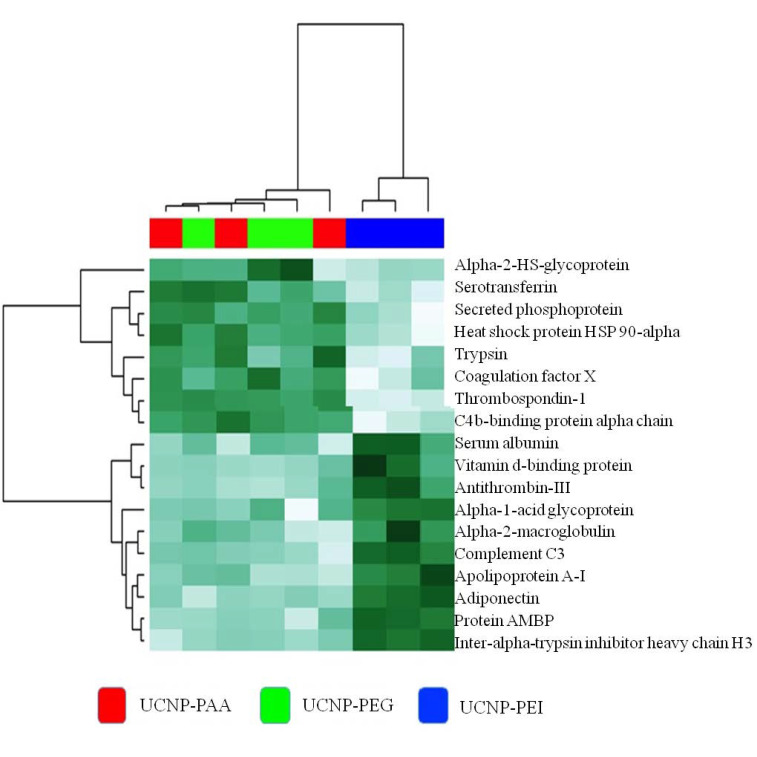
Heat map and hierarchical clustering of the protein abundances of UCNP-PAA, UCNP-PEG, and UCNP-PEI PC.

Multivariate statistical analysis using heatmap and hierarchical clustering highlighted different PC protein contents in UCNP-PEI, as shown in Figure 4. The heat map delineated 18 proteins abundant in the UCNP-PEI corona from the other abundant proteins detected in UCNP-PAA PC. The relative abundance among the different samples and within three replicates was easily observable. The heatmap showed good reproducibility of the hard PC bound to three different types of UCNPs. Cluster analysis showed the abundant proteins falling into two clusters: the upper cluster and the lower cluster. In the upper cluster, proteins were hardly found in UCNP-PEI corona, whereas those of the lower cluster were enriched in the PC extracted from UCNP-PAA-PC.

**Figure 5:**
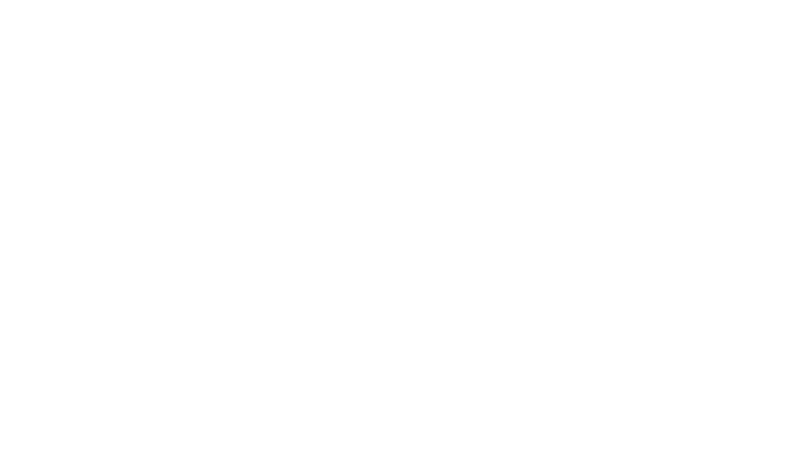
Box and whisker plots of the eight most-abundant proteins between the PC of the tested polymer-modified UCNP samples. The value of Log NSAF is plotted along the *y*-axis. The statistical significance was estimated using a Student’s *t*-test on the log-transformed NSAF values from the original biological triplicates (*p* < 0.05).

Figure 5 illustrates the normalised spectral abundance of the eight most frequently observed proteins identified from the hierarchical clustering. Thrombospondin-1, heat shock protein HSP90-alpha, and the C4b-binding protein alpha chain were more abundant in the UCNP-PAA PC, while inter-alpha-trypsin inhibitor heavy chain H2, adiponectin, alpha-2 microglobulin, complement c3, and apolipoprotein A-I were of higher abundance in the UCNP-PEI PC. The greater abundance of the complement C3 in PEI-coated NPs than in the other samples indicated that UCNP-PEI could trigger complement activation, which promoted phagocytosis and resulted in the clearance of nanoparticles from the bloodstream. A similar conclusion has been suggested elsewhere [18,40]. The data presented in Figure 5 showed that PAA and PEG coatings were likely to have similar bioeffects, while the response to PEI could be very different.

### 3.5. Evaluation of the Cell-UCNPs Interaction In Vitro: PC Effect on the Cellular Association of UCNPs

**Figure 6:**
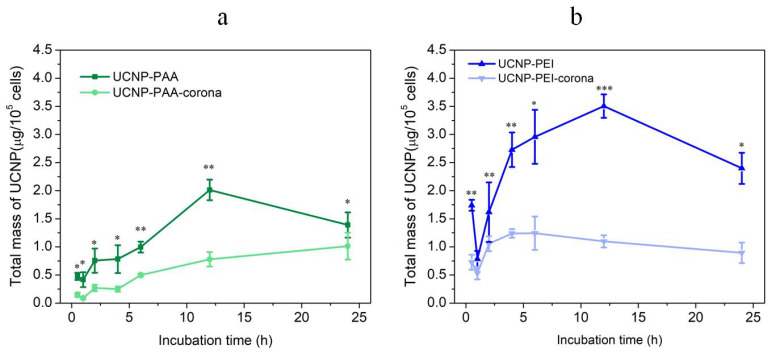
Kinetics of the bare and PC-coated UCNP-PAA (**a**) and UCNP-PEI (**b**) association with MDA-MB-231 cells as assayed by ICP-MS in terms of the total mass of UCNP taken at the concentration of 10 µg/mL per 10^5^ cells. Each value represents a mean of triplicate experiments ± standard deviation. Statistical significance was designated with * *p* < 0.05, ** *p* < 0.01, and *** *p* < 0.001.

It has been reported that NP uptake was initiated by adherence to the cell membrane and subsequent internalisation by cells via energy-dependent pathways. Lower adhesion usually resulted in decreased uptake efficiency [45]. NP’s association with cells, defined as both uptake and binding to cell membranes, was strongly dictated by NP size and surface properties. In order to gain insight into the interactions of the surface-modified UCNPs with cells, the association of MDA-MB-231 cells with bare and PC-preabsorbed UCNP-PAA-s and UCNP-PEI-s was evaluated at several timepoints by using inductively-coupled plasma mass-spectrometry (ICP-MS) multiphoton microscopy (MPM) techniques. MPM has been recognised as an attractive non-invasive approach to observe and analyse NP interaction with cells, including live cells. At the same time, quantitative evaluation of UCNP uptake and cell binding by MPM and other optical imaging modalities is prone to artefacts due to the detection threshold (in our case, evaluated as >10 UCNPs per pixel). Benchmarking the results of the UCNP cell association obtained by MPM against the results obtained by ICP-MS is deemed essential to assess the credibility of MPM quantitative measurements. The results of the cell association with UCNP polymer-coated with PEI and PAA and PC-preabsorbed assayed by ICP-MS are presented in Figure 6. Several important observations are summarised below.

Protein corona (PC) tended to reduce the cell binding and uptake (association) of both PAA- and PEI-coated UCNPs. This can be explained by noting the negative cytoplasm membrane charge of most cells, including MDA-MB-231 cells employed in this study. The more positive the surface charge of an NP, the stronger its adherence to the cell membrane and the stronger its association with the cell. Considering the surface charge measurement presented in Figure 2a, one can see that the PC rendered both PAA- and PEI-coated UCNPs more negative [40,46,47], thereby reducing their adherence to the cell membrane and hence reducing their cell association.

Using the same reasoning, one can explain the highest and lowest cell association of UCNP-PEI and UCNP-PAA-PC, respectively. The effect of the nearly neutral surface charge of UCNP-PEI-PC and UCNP-PAA (Figure 2a) was probably too weak to determine the cell adherence.

**Figure 7:**
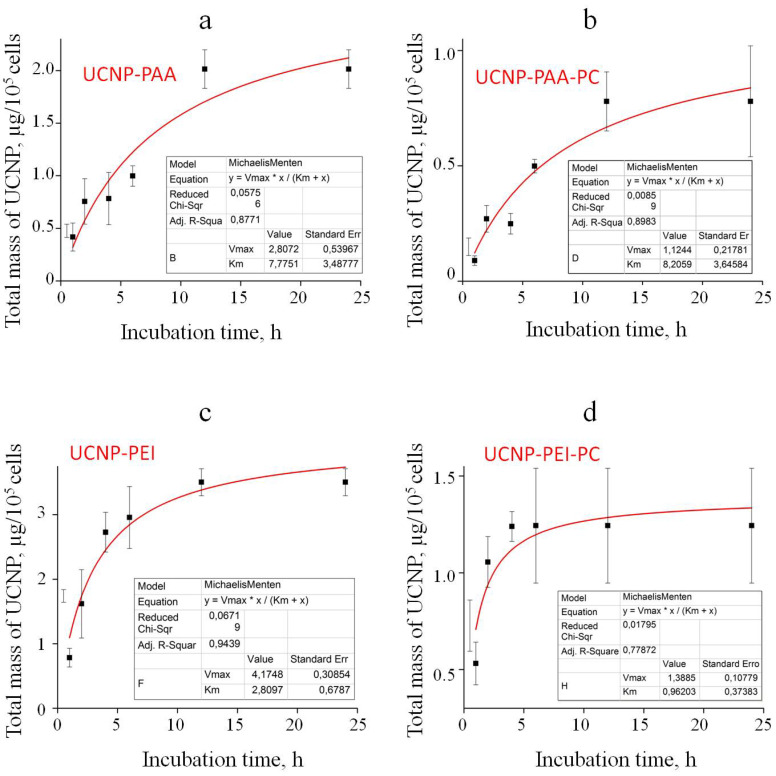
Plots of the kinetics of the UCNP association with MDA-MB-231 cells (data shown in Figure 6a) fitted with the Michaelis–Menten function for UCNP-PAA (**a**), UCNP-PAA-PC (**b**), UCNP-PEI (**c**), and UCNP-PEI-PC (**d**).

The kinetics of the UCNP cell association was similar, featuring 3 distinct phases: Phase 1, the amount of cell-associated UCNPs increased rapidly during an incubation period; Phase 2, the rate of the cell-associated UCNP increase slowed down; Phase 3, the increase rate reverted to a decrease rate (except for UCNP-PAA-PC sample). To gain more insight into the UCNP association kinetics, we fitted the experimental data in Phase 1 and Phase 2 with a Michaelis–Menten function commonly used to model drug uptake kinetics (Figure 7) [48]. The fitting trend indicated an active uptake process, such as endocytosis. Additionally, the decrease in the amount of cell-associated UCNPs in Phase 3 indicated that the exocytosis of UCNPs probably took place and had to be preceded by UCNP uptake. The UCNP uptake kinetics and conjectured exocytosis of UCNPs speaks in favour of the UCNP uptake process rather than binding and warrants further study.

Analysis of the confocal data shown in Figure S3a revealed no statistically significant differences in the uptake of UCNPs with or without PC. The results of the comparison of the UCNP binding and uptake are shown in Figure S3b, where no significant correlation was detected.

**Figure 8:**
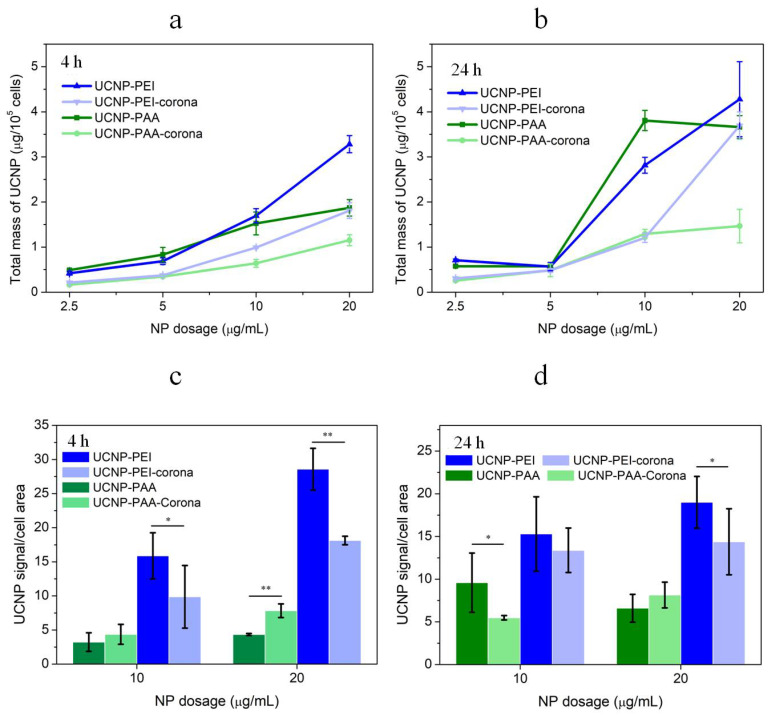
Average mass of UCNPs associated with MDA-MB-231 cells after 4 h (**a**) and 24 h (**b**) of incubation, characterised by ICP-MS (each value represents a mean of triplicate experiments ± standard deviation). Photoluminescence signal of UCNPs associated with MDA-MB-231 cells after 4 h (**c**) and 24 h (**d**) of incubation (the mean is characterised by mean photoluminescence signal intensity/cell area out of 100 cells in confocal images). * *p* < 0.05, ** *p* < 0.01.

ICP-MS was employed to assay one of the UCNP elemental constituents, yttrium (Y), as shown in Figure 8a,b plotted versus the concentrations of 2.5, 5, 10, and 20 µg/mL sampled 4 h and 24 h post incubation. The well-established ICP-MS provided a reliable quantitative evaluation of the UCNP content in the cells. Next, we analysed photoluminescent signals of UCNPs integrated over the MDA-MB-231 cell areas acquired by MPM, with the results are presented in Figure 8c,d. Unlike ICP-MS, the MPM method was prone to artefacts due to the detection limit. This implied that only UCNP medium or large clusters were detectable, in addition to high-density distributions of nanoparticles binned into one MPM image pixel. As such, the custom-designed MPM method provided a convoluted measure of the aggregation state and the amount of UCNPs. Further, owing to the nonlinear excitation of UCNPs, the photoluminescence signal was acquired from optical sections estimated as several tens of micrometers in thickness unlike conventional laser-scanning confocal microscopy capable of acquiring optical signals from optical sections of several micrometers. This implied that we were not able to differentiate between the cytoplasm-membrane-bound UCNPs and internalised UCNPs. The NPs uptake to MDA-MB-231 cells is size-dependent, with the highest cellular uptake reported to be ~250 nm NPs [49], exacerbating the ambiguity of the interpretation of MPM results. With this backdrop, the following observations can be made.

- The uptake and binding to MDA-MB-231 of both bare and PC-precoated UCNP-PAA and PEI measured by ICP-MS monotonically increased versus the dose at both incubation times of 4 h (Figure 8a) and 24 h (Figure 8b, the total mass values of UCNP-PAA at 10 and 20 mg/mL were statistically indifferent). The MPM results corroborated this trend at the 4 h timepoint (Figure 8c) but not at the 24 h timepoint (Figure 8d).
- Due to the highest positive surface charge, UCNP-PEI tended to exhibit the highest cell association. This tendency also transpired in the MPM measurements and was partly attributed to the aggregation tendency of UCNP-PEI, which enhanced the UCNP signal.
- The results obtained by both methods at the 24 h timepoint were less amenable for interpretation. We speculate that this was due to the cell active processing of UCNPs.

### 3.6. Evaluation of the Cell-UCNPs Interaction In Vitro: PC Effect on the UCNP Cytotoxicity

It is known that the cytotoxicity of UCNPs strongly depends upon the surface modification. The PC effect on the NP cytotoxicity was investigated using MDA-MB-231 cells. This cell line was chosen with the aim of prospective application of UCNPs in cancer theranostics. Cells at the exponential growth stage were incubated with serum-free DMEM in the presence of UCNP-PEI, UCNP-PAA, and the corresponding PC-modified samples. The absence of FBS in cell culture was first examined and showed no influence on the cell growth rate and cell morphology (Figure S4).

**Figure 9:**
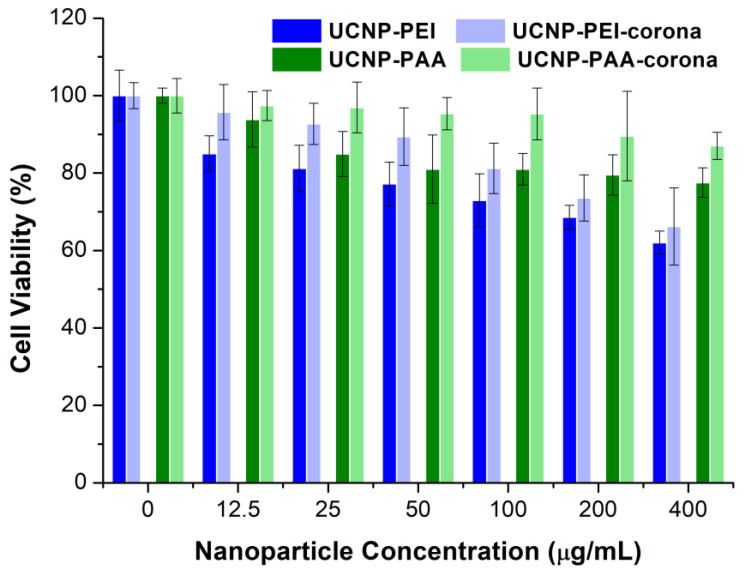
Cell viability data of MDA-MB-231 cells after treatment with polymer-coated and corresponding PC-modified UCNPs at concentrations ranging from 0 to 200 µg/mL for 24 h. Results of MTT assay, value format, mean ± standard deviation.

UCNP-PAA showed negligible influence on the MDA-MB-231 cells viability (viability remained > 80%) at concentrations from 0 to 200 µg/mL (Figure 9). In all tested UCNP concentrations, UCNP-PEI were more toxic than UCNP-PAA. UCNP-PEI had no effect on cell viability up to the concentration of 25 µg/mL, while the high concentrations (100 and 200 µg/mL) caused moderate cytotoxicity (survival rates less than 80%). Another study showed that PEI coating can both increase the toxicity of UCNPs and decrease it, depending on the choice of the cell line [50]. Here we obtain a slightly reduced cellular toxicity for both PC-modified samples, what may be the result of the lower rate of the cell association and uptake. The mitigation effect of the PC on the cell cytotoxicity was also observed in the reported studies using other NP types [18,25,51]. Therefore, the cytotoxicity of UCNPs can be mitigated by precoating nanoparticles with serum proteins to enable their safer application in biological systems.

**Figure 10:**
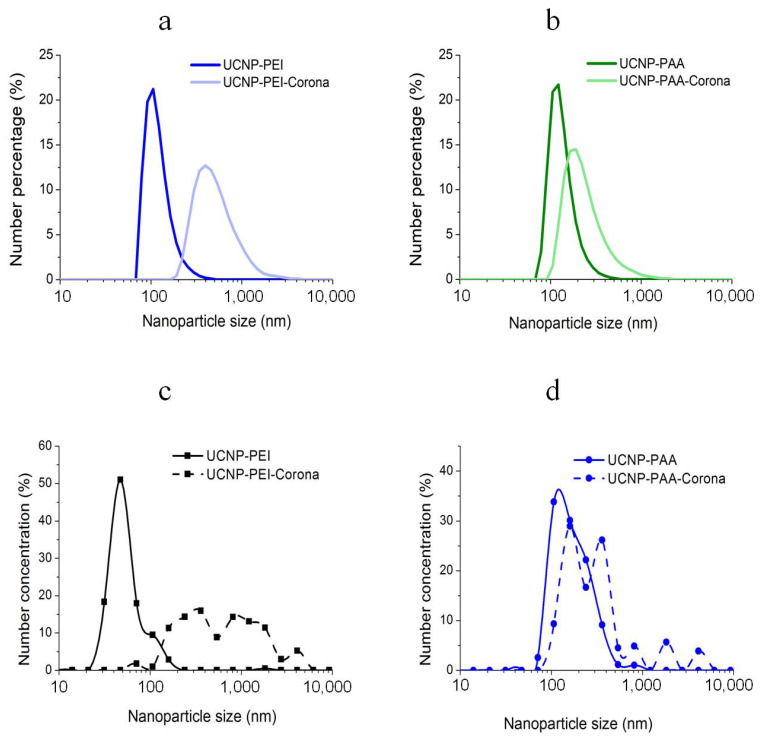
Hydrodynamic particle size distributions of aqueous dispersions of UCNPs (NaYF_4_: 20% Yb, 8% Tm) with PEI and PAA surface modifications and their hard PC-coated samples obtained by DLS (**a**,**b**) and FCS (**c**,**d**). Data for intact UCNPs are represented in a solid line, data for UCNPs modified with PC are dashed.

In order to demonstrate the potential of application of nanomaterials with nonlinear optical properties, we carried out direct observation of PC formation on polymer-coated UCNPs in protein solutions by means of correlation spectroscopy of UCNP emission. To demonstrate its adequate performance in clear solutions, we carried out a side-by-side comparison of the PSDs of bare and hard-PC-acquired polymer-coated UCNP colloids captured by DLS and FCS, with the results presented in Figure 9. All measurements were carried out in clear aqueous solutions. PSDs of bare UCNP-PEI and UCNP-PAA colloids measured by DLS were comparatively narrow, peaking at approximately 100 nm (Figure 10a,b, solid lines), while PSDs of the same colloids measured by FCS featured broader PSDs. PSD of bare UCNP-PEI featured two peaks centred at 50 nm and 100 nm, while the PSD of bare UCNP-PAA featured one peak at 100 nm, with a shoulder extending to ca 500 nm. This indicated that FCS could report more polydisperse PSD distributions than DLS. It is known that DLS is prone to underestimation of the sample polydispersity. The PC effect was manifested by an upshift of the PSDs of all colloidal samples and was adequately captured by both FCS and DLS. The PSD upshift was significant in the case of hard-PC-acquired UCNP-PEI colloids measured by DLS and was more profound in the case of FCS measurement. We interpreted that an increase of the hydrodynamic diameter of UCNPs eventually led to the formation of large UCNP-PC aggregates. In general, the FCS results were in reasonable agreement with PSD measured by DLS, while the FCS method captured the sample polydispersity more adequately and showed promise for the direct measurement of PC formation.

## 4. Conclusions

Formation of protein corona (PC) on the surface of NPs changes their biological identity to govern the NP interactions and requires better understanding of the key underlying processes. Upconversion nanoparticles (UCNPs) appeared to be an attractive NP model due to their unique optical properties, which afforded direct observation and analysis of PC formation in biological fluids. We investigated the composition of the hard PC and its formation kinetics using UCNPs coated with positively and negatively charged polymers implemented by polyethyleneimine (PEI) and polyacrylic acid (PAA), respectively.

The hydrodynamic diameter of polymer-coated UCNPs increased at the completion of the PC formation. This observation was consistent with an assayed increase of the protein content, where UCNP-PEI-PC adsorbed a ~4-times greater amount of serum proteins than UCNP-PAA-PC, and their PC composition varied considerably. The PC was found to modulate the cell binding and uptake, although the surface charge of the UCNP polymer coating appeared to be the main determinant of these interactions, especially in the case of UCNP-PEI. The cytotoxicity of the UCNP-polymer-PC was reduced for both PEI and PAA polymer coatings. As a proof-of-principle of the value added by UCNP as the nanoparticle model, we successfully demonstrated the direct size measurement of UCNP-polymer and UCNP-polymer-PC in clear aqueous and turbid serum colloidal solutions by means of fluorescence correlation spectroscopy.

We believe that the reported systematic study of the interaction between serum proteins and NPs can help design drug carriers for prolonged circulation, lower toxicity, and enhanced targeting effect to achieve therapeutic and diagnostic benefits.

## Supplementary Materials

The following supporting information can be downloaded at: https://www.mdpi.com/article/10.3390/cells11223644/s1, Figure S1: TEM images (a), FTIR spectra (b) and TG analyses (c) of UCNP-PEI and UCNP-PAA. Upconversion photoluminescence spectra of UCNP-PEI and UCNP-PAA dispersed in distilled water at the concentration of 1 mg/mL (d); Figure S2: DLS measurement of proteins in complete cell culture medium; Table S1: List of the 20 most abundant proteins detected in the corona binding UCNPs by nanoLC-ESI-MS mass spectrometry. The isoelectric points were calculated using the protein analysis tool Compute pI/Mw on the ExPASy server (http://web.expasy.org/); Figure S3: Photoluminescence signal of the bare and PC-coated UCNP-polymer nanoparticles associated with MDA-MB-231 cells assayed by photoluminescence laser-scanning microscopy in terms of the PL signal intensity per 100 cell area (a). Mass of the bare and PC-coated UCNP-polymer nanoparticles after binding and uptake by MDA-MB-231 cells estimated by ICP-MS (b). Each value represents a mean of triplicate experiments ± standard deviation; Figure S4: Phase-contrast images of MDA-MB-231 cells growing in serum-free DMEM and complete cell culture medium (a). Cell viability of MDA-MB-231 cells after incubation with complete cell culture medium and serum-free DMEM for 24 h (b).
