# Supplementary material for: The Surface Charge of Polymer-Coated Upconversion Nanoparticles Determines Protein Corona Properties and Cell Recognition in Serum Solutions
Source: Cells. 2022 Nov 17;11(22):3644. doi: 10.3390/cells11223644 (PMC9688575; doi:10.3390/cells11223644)
Supplement: Supplementary file 1 [file cells-11-03644-s001.zip › cells-1957373-supplementary.pdf]

Supporting Information

The surface charge of polymer-coated upconversion nanoparticles determines protein corona properties and cell recognition in serum solutions

Liuen Liang [1], Arun V. Everest-Dass [1], Alexey B. Kostyuk [2], Zahra Khabir [1,3], Run Zhang [4], Daria B. Trushina [5,6,*], Andrei V. Zvyagin [1,2,5,7]

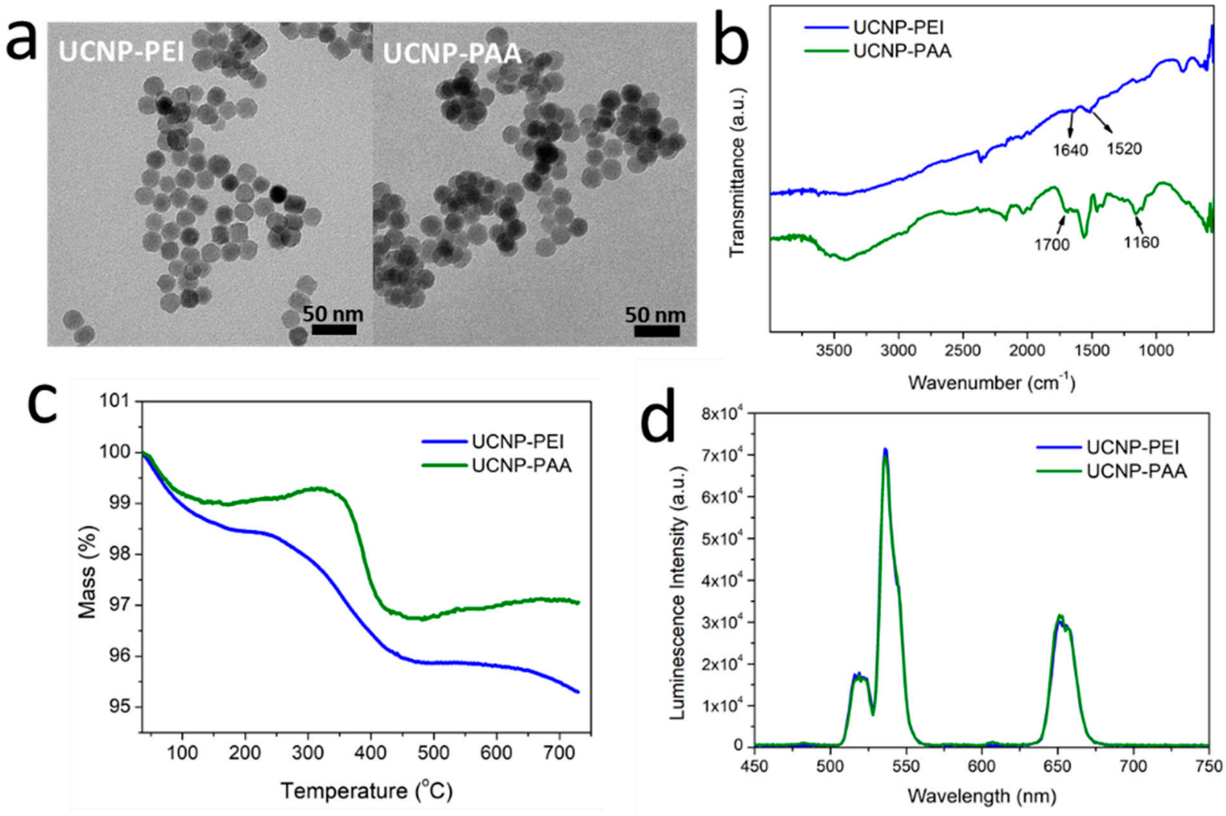

Figure. S1. TEM images (a), FTIR spectra (b) and TG analyses (c) of UCNP-PEI and UCNP-PAA. Upconversion photoluminescence spectra of UCNP-PEI and UCNP-PAA dispersed in distilled water at the concentration of 1 mg/mL (d).

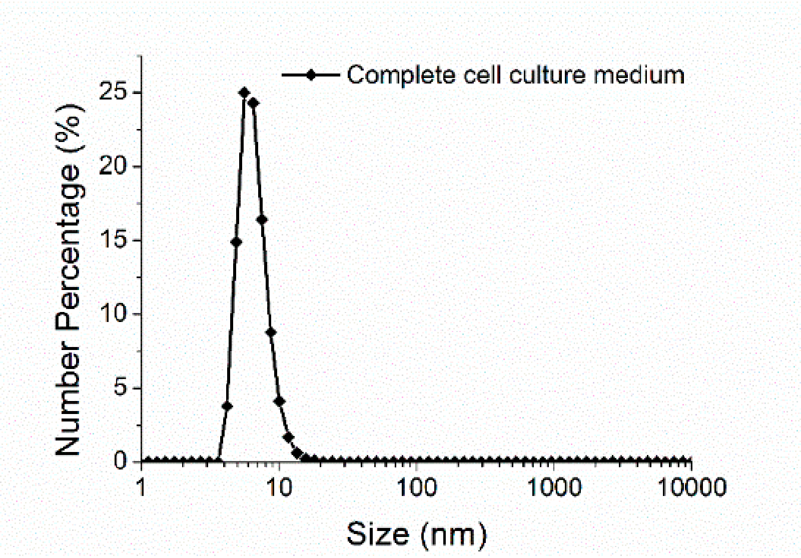

Figure. S2. DLS measurement of proteins in complete cell culture medium.

Table. S1 List of the 20 most abundant proteins detected in the corona binding UCNPs by nanoLC-ESI-MS mass spectrometry. The isoelectric points were calculated using the protein analysis tool Compute pI/Mw on the ExPASy server (http://web.expasy.org/).

No.	Protein	Isoelectric point (pI)	Molecular weight (Da)
1	Alpha-2-HS-glycoprotein	5.26	38394
2	Serum albumin	5.82	69248
3	Platelet factor	6.11	9517
4	Apolipoprotein A-II	7.80	11195
5	Hemoglobin fetal subunit beta	6.51	15849
6	Alpha-1-antiproteinase	6.05	46075
7	Apolipoprotein A-I	5.71	30258
8	Tetranectin	5.47	22130
9	Prothrombin	5.97	70461
10	Apolipoprotein E	5.67	36003
11	Protein AMBP	7.81	39209
12	Adiponectin	5.44	26117
13	Platelet factor 4	8.46	9124
14	Inter-alpha-trypsin inhibitor heavy chain H3	5.59	99489
15	Transthyretin	5.90	15717
16	Thrombospondin-1	4.74	129451
17	Secreted phophoprotein 24	8.30	23119
18	C4b-binding protein alpha chain	5.98	68841
19	Alpha-1-acid glycoprotein	5.62	23168
20	Hemoglobin subunit alpha	7.94	15528

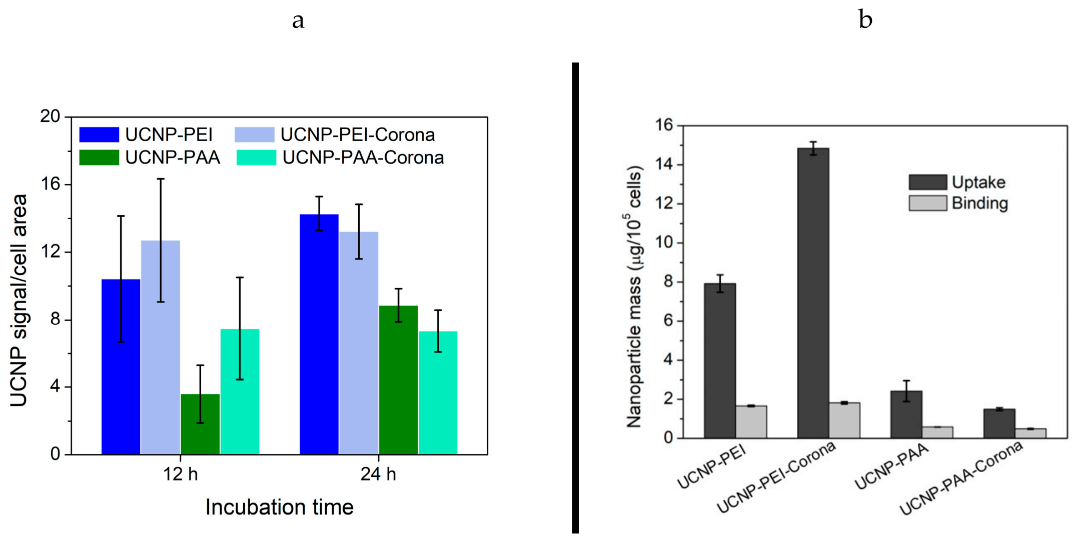

Figure S3. Photoluminescence signal of the bare and PC-coated UCNP-polymer nanoparticles associated with MDA-MB-231 cells assayed by photoluminescence laser-scanning microscopy in terms of the PL signal intensity per 100 cell area (a). Mass of the bare and PC-coated UCNP-polymer nanoparticles after binding and uptake by MDA-MB-231 cells estimated by ICP-MS (b). Each value represents a mean of triplicate experiments ± standard deviation.

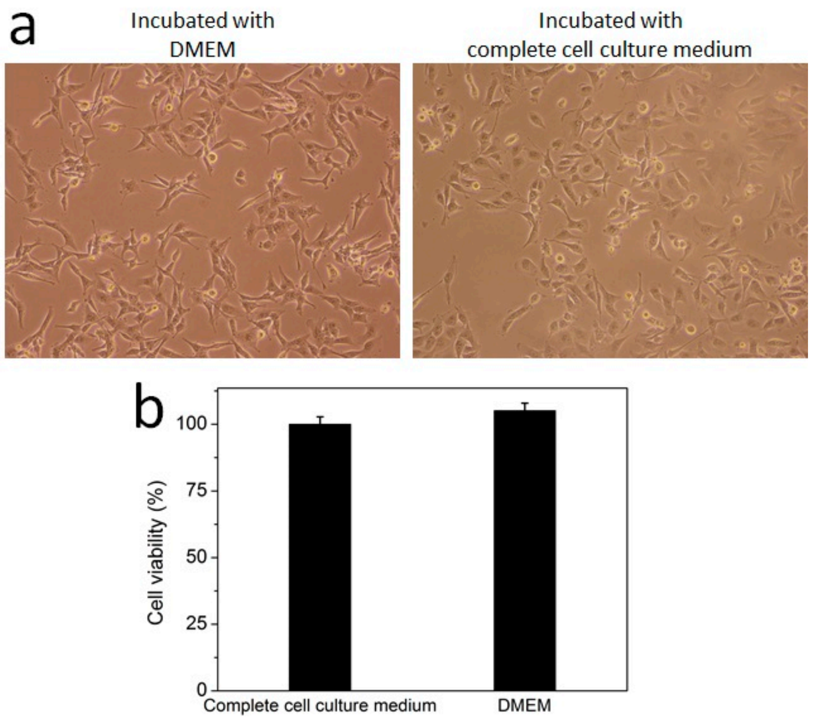

Figure S4. Phase-contrast images of MDA-MB-231 cells growing in serum-free DMEM and complete cell culture medium (a). Cell viability of MDA-MB-231 cells after incubation with complete cell culture medium and serum-free DMEM for 24 h (b).
